# Efficacy and Mid-Term Outcomes of Paclitaxel-Coated Balloon (Optilume^®^) for Penile Strictures

**DOI:** 10.3390/jcm14176022

**Published:** 2025-08-26

**Authors:** Johannes Salem, Juan Jose Menendez-Suarez, Georgi Tosev, Hendrik Borgmann, Timur Kuru

**Affiliations:** 1 Department of Urology, Faculty of Health Sciences Brandenburg, Brandenburg Medical School Theodor Fontan, 14770 Brandenburg, Germany; johannes.salem@mhb-fontane.de (J.S.); hendrik.borgmann@mhb-fontane.de (H.B.); timur.kuru@mhb-fontane.de (T.K.); 2 CUROS Urology Center, 50996 Cologne, Germany; 3 Department of Urology, St Elisabeth Hospital Cologne Hohenlind, 50935 Cologne, Germany; 4 Praxis Urologie Mannheim, 68239 Mannheim, Germany; dr.tosev@yahoo.com; 5 Department of Urology, University Hospital Heidelberg, Im Neuenheimer Feld 420, 69120 Heidelberg, Germany

**Keywords:** penile urethral stricture, minimally invasive treatment, drug-coated balloon, urethral stenosis, mid-term outcomes, urethroplasty alternative

## Abstract

**Background/Objectives:** Penile urethral stricture is a therapeutically challenging condition that significantly impacts quality of life and is often managed initially with urethral dilation or internal urethrotomy. However, both techniques are associated with high recurrence rates, limited long-term efficacy, and potential adverse effects, particularly in the penile urethra. Urethroplasty remains the gold standard but is invasive and not suitable for all patients. Optilume, a paclitaxel-coated balloon, combines mechanical dilation with localized drug delivery to reduce recurrence rates and the need for re-intervention. This study evaluated its effectiveness in patients with penile urethral strictures. **Methods:** A retrospective, multicenter study was conducted at two German clinics. Eight male patients (mean age 59) with symptomatic penile urethral strictures underwent Optilume treatment. Symptom severity was assessed using the International Prostate Symptom Score (IPSS) and quality of life (QoL) scores before and after treatment. The primary endpoint was symptom improvement, while the secondary endpoint was the need for reintervention. Patients were followed for a median of 16.5 months. Statistical analyses included Wilcoxon signed-rank and Mann–Whitney U tests. **Results:** The median IPSS improved from 25.5 to 5.0 and QoL scores from 4.5 to 1.0 after treatment (*p* < 0.01 for both). No patients required reintervention during follow-up. The subgroup analysis showed slightly better outcomes in patients without prior interventions, although differences were not statistically significant. The stricture length did not correlate with treatment response. **Conclusions:** Optilume significantly reduces urinary symptoms and improves QoL in penile urethral strictures, and the absence of re-interventions during follow-up underscores its durable mid-term success. It offers a minimally invasive alternative to urethroplasty, particularly for patients seeking symptom relief with a shorter recovery time and no hospital stay or general anesthesia. These preliminary findings suggest that Optilume may be a promising minimally invasive option for selected patients. Larger, controlled studies are warranted to validate these results and refine patient selection criteria.

## 1. Introduction

The male urethra can be anatomically and histologically divided into anterior and posterior segments. The anterior urethra comprises the penile and bulbar regions, both enveloped by the corpus spongiosum [1,2]. In men, a urethral stricture represents a focal constriction of the anterior urethra resulting from fibrotic scarring of the urothelial lining and surrounding spongiosal tissue [1,3]. Urethral strictures occur in approximately 0.23% to 0.63% of men over the course of their lives [2,4].

Historically, endoscopic techniques such as direct vision internal urethrotomy (DVIU) and urethral dilation have served as first-line treatments because they are cost-effective and widely familiar to urologists. However, these approaches are burdened by high rates of recurrence, which vary according to stricture length, location, and etiology. Open urethroplasty remains the gold standard for durable stricture management, with long-term success rates reported to range between 79.3% and 90.4% [5,6,7]. Nonetheless, urethroplasty is more invasive, carries greater perioperative morbidity, and typically necessitates general anesthesia [1].

Steenkamp and colleagues found that urethral dilation and direct vision internal urethrotomy (DVIU) yield similar success rates, although both techniques become less effective as the stricture length increases, with success falling from about 60 percent in shorter lesions to under 40 percent in longer ones [8]. Reported recurrence rates following DVIU span from 23.3 percent [9] and 48.4 percent [10] to 86.7 percent after up to three procedures [11] and 91.7 percent in recent series with at least one year of follow-up [12]. In most cohorts, the median interval to recurrence is under twelve months [8,9,10,11,12].

Given the similar complication profiles and limited long-term efficacy of both dilation and DVIU, neither is clearly superior. Graversen and co-workers noted an elevated risk of erectile dysfunction when strictures involve the penile urethra in addition to poor patency rates [13], leading the European Association of Urology guidelines to recommend against DVIU in that region [14]. Additionally, if urethroplasty is a feasible option, further dilation or DVIU attempts are discouraged, since repeated endoscopic interventions not only have diminishing returns but may also compromise subsequent surgical outcomes. The EAU guidelines, therefore, advise limiting endoscopic management to two attempts at most, to avoid delaying definitive urethroplasty and exacerbating stricture complexity [14]. Recently, a novel dilation technique utilizing a paclitaxel-coated balloon (Optilume) has been introduced. By combining mechanical urethral dilation with the anti-fibrotic and anti-proliferative properties of paclitaxel, this approach has yielded encouraging outcomes. Initial ROBUST I [15] and ROBUST III [16] trials reported that the procedure is safe, can be performed on an outpatient basis, and maintains low complication rates over long-term follow-up periods ranging from three years [16] to five years [15]. The pathophysiology of urethral stricture formation is multifactorial and involves a complex interplay between mechanical injury, inflammation, and aberrant wound healing. Paclitaxel, a cytotoxic agent widely used in oncology and vascular interventions, acts by stabilizing microtubules and inhibiting cellular proliferation. In the context of urethral strictures, its antiproliferative effects are hypothesized to reduce the risk of stricture recurrence by limiting scar formation and smooth muscle hyperplasia. The use of drug-coated balloons for localized paclitaxel delivery effectively reduces systemic exposure and minimizes associated adverse effects [17], and has demonstrated success in treating in-stent restenosis in vascular medicine [18].

The translation of this concept to urology represents an innovative leap that aims to preserve the urethral caliber while avoiding the morbidity associated with repeated endoscopic interventions or open surgery. This minimally invasive approach is particularly appealing in anatomically sensitive areas such as the penile urethra, where preserving erectile function and minimizing tissue trauma are critical considerations.

In evaluating urethral stricture treatments, there is no universally accepted definition of “success.” According to the European Association of Urology (EAU), success remains a subjective and poorly standardized concept. While urethral patency—either confirmed objectively by imaging or calibration, or subjectively by symptom relief—is often used as a surrogate endpoint, it does not necessarily reflect the patient’s perception of treatment outcome. Functional consequences such as post-void dribbling, ejaculatory dysfunction, or altered genital appearance may persist even in anatomically patent urethras [16] Therefore, the EAU panel explicitly discourages the use of the term “success” and instead recommends using more objective parameters such as “patency rate” or “stricture recurrence rate,” based on the absence of reintervention or clinically significant symptom recurrence. Moreover, if the patient reports satisfaction with the treatment and is free of symptoms, performing invasive follow-up studies such as cystoscopy or retrograde urethrography has little clinical relevance. Detecting a radiographic or endoscopic narrowing in an asymptomatic patient would not alter the therapeutic course, as no additional intervention would be warranted in the absence of functional impairment. Additionally, these diagnostic procedures are not risk-free; they carry potential adverse effects including urinary tract infections, hematuria, and iatrogenic trauma, which in some cases may even promote new stricture formation. In this context, our study prioritizes patient-reported outcomes and the absence of reintervention as more meaningful and patient-centered indicators of treatment durability. Importantly, anatomical narrowing of the urethra does not always correlate with clinical significance. It is generally accepted that a urethral stricture tends to impair urinary flow once the luminal caliber falls below 10 French [19]. However, evidence suggests that many radiographically or endoscopically detected strictures—particularly those exceeding 16 Fr—remain stable and asymptomatic [19]. In a prospective observational study by Purohit et al., 42 incidentally discovered, subclinical strictures (>16 Fr) were followed with periodic cystoscopic evaluations over a median of 23 months. Only five cases (12%) showed any progression to a narrower caliber (11–15 Fr), and none of the patients developed symptoms or required surgical intervention. Similarly, Erickson et al. analyzed cases of anatomic recurrence (defined as <16 Fr) following urethroplasty and found that only 65% of patients reported symptoms [20]. Interestingly, many asymptomatic individuals declined further procedures, citing significant subjective improvement after their initial treatment. These cases were, thus, considered functional “successes,” despite the presence of anatomical narrowing [20]. Such findings reinforce the notion that routine invasive follow-up in asymptomatic patients may lead to overtreatment and unnecessary morbidity.

## 2. Materials and Methods

We performed a single-arm, retrospective, multicenter, nonrandomized, open-label investigation at two German clinics to evaluate the effects of the Optilume^®^ drug-coated balloon (DCB) on lower urinary tract symptoms (LUTS) and related morbidity in patients with penile urethral strictures. Symptom severity was quantified using the International Prostate Symptom Score (IPSS) and quality of life (QoL) metrics, assessed both before treatment and at follow-up.

Men aged 18 years or older presenting with symptomatic penile urethral strictures confirmed by cystoscopy or retrograde urethrography were enrolled. The baseline characteristics of the study population are summarized in Table 1. The primary outcome measures were changes in IPSS and QoL scores following treatment. The secondary outcome was duration of intervention-free survival. Statistical analyses were conducted with IBM SPSS Statistics version 29.0 (2022), and a two-tailed *p* value below 0.05 was considered statistically significant. All participants provided written informed consent, and the study received approval from the institutional ethics committee (approval number F-2023-048).

In both centers, all Optilume^®^ procedures were performed by experienced urologists using a harmonized protocol under either deep sedation (propofol infusion) or a combination of intravenous metamizole (2.5 g) and loco-regional urethral anesthesia. Prophylactic antibiotics were started 48 h before and continued for 72 h after the intervention.

After obtaining baseline retrograde urethrograms to characterize stricture length and morphology, a 20 Fr rigid cystoscope (Karl Storz or Richard Wolf, 0° lens) was introduced and a 150 cm hydrophilic guidewire (Radifocus^®^ Terumo) was carefully negotiated through the stenotic segment. In strictures abutting the meatus, where in situ balloon hydration can be challenging, the Optilume balloon is first immersed in 0.9% saline for 60 s to ensure complete drug coating activation.

**Figure 1:**
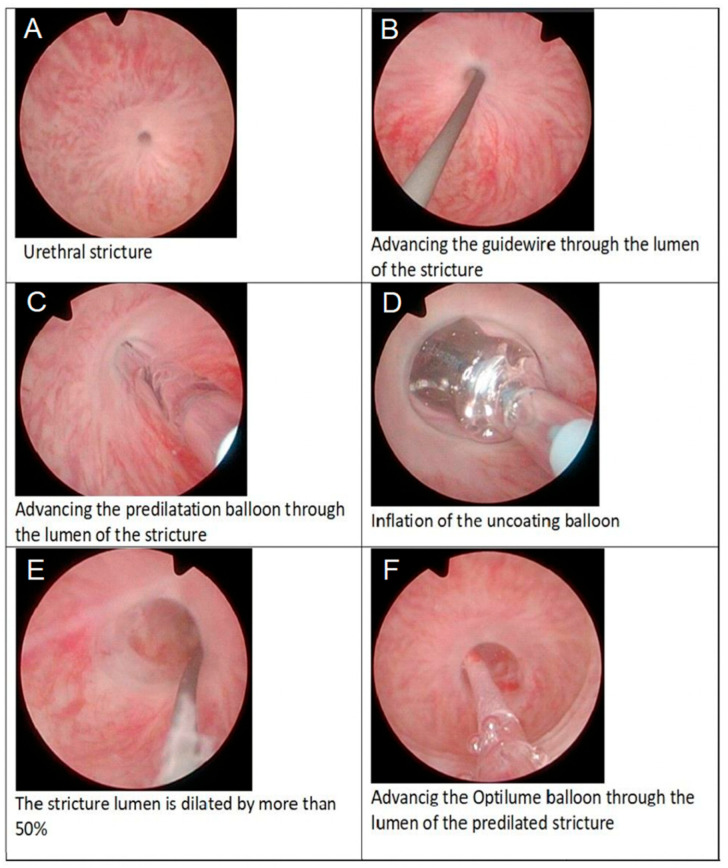
Endoscopic sequence of Optilume® balloon urethroplasty for recurrent urethral stricture: (**A**) identification of the stricture, (**B**) advancement of the guidewire through the narrowed lumen, (**C**) passage of the predilation balloon over the wire, (**D**) inflation of the uncoated predilation balloon, (**E**) achievement of >50 % luminal enlargement, and (**F**) introduction of the drug-coated Optilume® balloon into the dilated segment for final therapeutic dilation.

**Figure 2:**
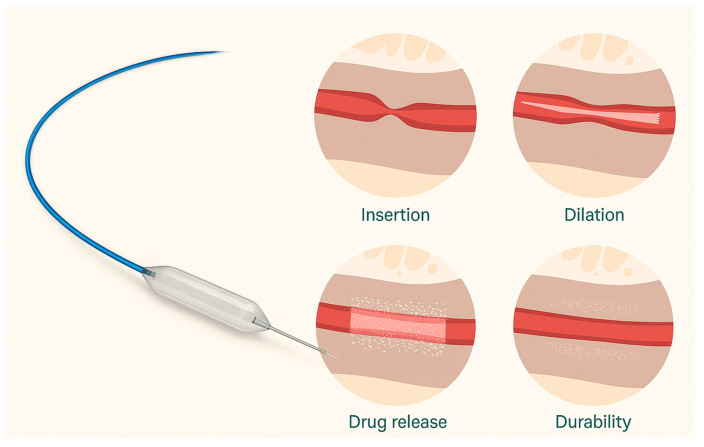
**Left**: The Optilume paclitaxel-coated balloon device featuring a specialized balloon catheter with a blue guidewire and drug-coated surface. **Right**: Sequential illustration of therapeutic phases. (1) Insertion: Initial placement of the deflated balloon in the strictured area. (2) Dilation: Mechanical expansion of the strictured segment. (3) Drug release: Transfer of paclitaxel coating into the surrounding tissue, (4) Durability: Maintenance of luminal patency following treatment, with a sustained drug effect in tissue. Adapted from Ref. [21].

A 24 Fr uncoated balloon (UroMax^®^) is then advanced over the guidewire and inflated to achieve approximately 50% luminal gain. If cystoscopic inspection reveals no significant mucosal tears or bleeding, the uncoated balloon is exchanged for the Optilume drug-coated balloon (5 cm length, 30 Fr diameter), which is inflated to 10 atm for at least 7 min. In cases of notable hemorrhage during predilatation, the 24 Fr balloon is retained for the remainder of the procedure to reduce risk. Finally, a 14 Fr Foley catheter is left indwelling for 48 h to ensure adequate urinary drainage and mucosal apposition. As illustrated in Figure 1 and Figure 2, the procedure entails guidewire placement, sequential predilation, and final drug-coated balloon dilation to restore urethral patency. As per current guideline philosophy and in line with EAU recommendations, no routine anatomical follow-up was performed in asymptomatic patients; instead, follow-up focused on patient-reported symptom relief, absence of retreatment, and overall satisfaction rather than routine anatomical assessment.

## 3. Results

### 3.1. Study Population

Eight patients with clinically significant penile urethral strictures were enrolled, with a mean age of 61 years (range 47 to 79). Four strictures were iatrogenic and four were idiopathic. All patients completed a clinical visit with IPSS questionnaire prior to treatment and again in January 2025, after a minimum follow-up period of 14 months. The median pretreatment International Prostate Symptom Score was 26, and six of eight patients (75 percent) reported severe lower urinary tract symptoms (IPSS > 19). The average quality of life score before treatment was 5. Four patients had not undergone any prior intervention. Among the remaining four, two had rigid dilation performed 12 and 8 months before enrollment, one had balloon dilation 10 months prior, and one had both direct vision internal urethrotomy 18 months earlier and rigid dilation 6 months before inclusion. Stricture lengths ranged from 1.5 to 8.0 cm (mean 3.4 cm). Patients were followed for an average of 16.5 months (range 14 to 43) without requiring further intervention.

### 3.2. Impact of Optilume on IPSS and QoL

Treatment with Optilume resulted in significant improvements in both urinary symptoms and quality of life, as summarized in Table 2. The median pre-treatment IPSS was 25.5 (range: 14–28), which improved to 5.0 (range: 1–17) following treatment. This translates to a median percentage improvement of 48.6%, showing a substantial alleviation of symptoms.

Similarly, QoL scores demonstrated significant improvement. The median pre-treatment QoL score was 4.5 (Range: 4–5), which decreased to 1.0 (range: 1–4) post-treatment. The median percentage improvement in QoL was 50.0%, reflecting a marked improvement in patients’ perceived quality of life. The statistical analysis using the Wilcoxon signed-rank test confirmed that these improvements in both IPSS and QoL were statistically significant (*p* < 0.01).

### 3.3. Sub-Analysis by Number of Prior Interventions

To assess differences in outcomes, patients were grouped into two categories: those without prior interventions (*n* = 4) and those who had undergone at least one prior intervention (*n* = 4).

Patients with no history of prior interventions showed a good improvement after treatment. Their median pre-treatment IPSS was 21.5 (range: 14–26), which improved to 5.0 (range: 3–7) post-treatment. The median percentage improvement in IPSS for this group was 48.6%. QoL scores also improved significantly, with a median pre-treatment score of 4.0 (range: 4–5), reducing to 1.0 (range: 1–2) post-treatment, yielding a median percentage improvement of 50.0%.

Patients with at least one prior intervention also benefited from the treatment. Their median pre-treatment IPSS was 26.5 (range: 21–28), which improved to 8.5 (range: 1–17) post-treatment. The median percentage improvement in IPSS was 45.7%. In terms of QoL, scores improved from a median of 5.0 (range: 4–5) pre-treatment to 1.5 (range: 1–4) post-treatment, with a median percentage improvement of 50.0%.

Both groups achieved meaningful improvements, and the difference in IPSS improvement between the two was not statistically significant (*p* = 0.09, Mann–Whitney U test). Similarly, the difference in QoL improvement did not reach statistical significance (*p* = 0.07), suggesting that the outcome is not affected by the number of prior treatments.

### 3.4. Sub Analysis by Stricture Length

To investigate the relationship between stricture length and treatment effectiveness (measured by improvements in IPSS and QoL scores), a Pearson correlation analysis was performed. The correlation between the stricture length and IPSS and QoL improvement was not statistically significant (IPSS *p* = 0.27 and QoL *p* = 0.22).

These findings suggest that within this sample, the stricture length did not exhibit a statistically significant relationship with the degree of improvement in IPSS or QoL scores following treatment. Larger sample sizes or the inclusion of additional covariates may be required to better understand potential relationships. These findings should be interpreted with caution due to the small sample size, which limits the statistical power of the correlation analysis.

### 3.5. Follow-Up and Re-Intervention Rates

There was no reintervention needed in the follow-up period, which had a median duration of 16.5 months (range: 14–43). All strictures were successfully resolved with a single Optilume treatment, demonstrating its mid-term efficacy and durability of the procedure.

## 4. Discussion

These results demonstrate that Optilume is effective in significantly reducing urinary symptoms and improving quality of life in patients with penile urethral strictures.

According to the currently available Robust I and III studies, functional success in the treatment of urethral strictures was achieved in 71% of patients at 3 years in the Robust III study [22] and in 58% of patients at 5 years in the Robust I study [15]. However, specific data on penile strictures were not reported separately in the published results.

A recent scoping review by Gauhar et al. summarized the current evidence on Optilume, confirming its mid- to long-term efficacy and safety in anterior strictures overall, but also emphasized the lack of robust data in the penile urethra [23]. More specifically, Oszczudłowski et al. highlighted that only eight patients with penile strictures were included in the ROBUST III trial, with freedom from reintervention rates dropping from 75% at 1 year to 47% at 2 years, underscoring the uncertainty regarding long-term durability in this anatomical location [24]. Importantly, all patients included in ROBUST III had undergone multiple prior endoscopic interventions, which may have negatively influenced treatment durability and limits the generalizability of these results to treatment-naïve populations.

Urethroplasty is considered the gold standard for treatment of penile strictures, with long-term success rates ranging from 72% [25] to 80% [6]. Penile strictures are associated with a higher risk of recurrence when treated with endoscopic techniques such as urethrotomy, which achieve long-term success rates of only 20–30%. Urethrotomy is generally considered a temporary solution, and repeated procedures may result in more complex strictures and a hostile tissue environment, thereby complicating any future urethroplasty and making it more challenging to perform [26]. A direct comparison between Optilume, urethrotomy, and urethroplasty would be valuable to clarify the role of this new technology. Current guidelines already recommend early transition to urethroplasty if urethrotomy fails [14].

The patients in our study without prior interventions experienced slightly better outcomes, suggesting that early use of Optilume may offer the greatest benefits. However, this difference was not statistically significant, possibly due to the small cohort size. Although the results should be interpreted with caution, this trend supports the rationale for conducting larger, prospective studies. The absence of re-interventions during the follow-up period underscores the durability and mid-term success of Optilume treatment. Further advantages of Optilume include its significantly shorter learning curve compared to urethroplasty and its feasibility in an outpatient or office-based setting. The procedure is faster, does not require general anesthesia, and involves a considerably shorter catheterization time. While Optilume provides good mid-term results with minimal invasiveness, it is currently a more expensive option, with the balloon costing approximately 2650 Euros in Germany. Future research should further investigate the impact of prior treatments on outcomes to optimize patient selection and refine treatment strategies. Optilume remains a valuable alternative for patients with penile strictures who are either unfit or unwilling to undergo urethroplasty or those seeking rapid symptom relief with favorable mid-term results.

For penile or pendulous urethral strictures, the balloon diameter should be chosen to closely match the caliber of the healthy distal urethra. In the ROBUST III study, balloon sizes for penile strictures were roughly evenly divided between 24 F and 30 F Optilume DCBs [22]. In the peer-reviewed ROBUST trials (I–III), no cases of urethral dissection or true perforation were reported with the Optilume DCB. Instead, the most frequently observed adverse events were urinary tract infections, post-procedural hematuria, dysuria, and acute urinary retention [15,22]. A recent comprehensive review also found no serious mechanical complications such as dissection or perforation in nearly 300 treated strictures [25]. We use a 24 F predilatation balloon; if no significant urethral injury was observed, we then proceed with the 30 F DCB. However, if notable bleeding occurred after predilatation, we retained the 24 F size.

In our cohort, Optilume^®^ DCB dilation was not associated with any serious mechanical complications or new-onset erectile dysfunction, but several potential risks warrant discussion. First, urethral dissection may occur when high-pressure radial expansion creates a submucosal “false passage”, particularly in densely fibrosed segments. Second, we observed no cases of urethral perforation in our series. Although rare, this serious complication can occur if balloon expansion pressures exceed tissue tolerance or if undetected fibrotic defects are traversed. Nonetheless, operators should remain vigilant for contrast extravasation or sudden balloon pressure loss; recommended management includes immediate catheter removal, prolonged drainage, and delayed repeat imaging before further intervention. Finally, although inflation of a 30 F balloon raises theoretical concerns about injury to the corpora cavernosa and subsequent erectile dysfunction, neither the ROBUST trials nor a recent systematic review of nearly 300 Optilume treatments reported any such events [27]. Van Dyke et al. further demonstrated stable IIEF-5 scores at two years (baseline 24.3 ± 1.2 vs. 23.9 ± 1.5; *p* = 0.28) [28].

In our study, the absence of reintervention during follow-up aligns with mid-term outcomes from the ROBUST trials and reinforces the therapeutic potential of Optilume as a standalone treatment. Notably, the improvements in both IPSS and QoL scores suggest not only symptom relief but also a meaningful enhancement of patient well-being. This dual impact is essential when evaluating the clinical value of novel interventions, especially in conditions like urethral strictures that can significantly impair daily functioning and mental health.

Patient selection remains a key determinant of success in stricture management. In our cohort, individuals without prior interventions experienced slightly better outcomes, hinting at the possibility that early adoption of Optilume may yield superior results. This notion is consistent with the concept of minimizing cumulative trauma to the urethral epithelium and surrounding tissues. Repeated instrumentation is known to induce further fibrosis, potentially transforming a simple stricture into a more complex and resistant lesion. Thus, integrating Optilume earlier in the treatment algorithm could preempt this progression and preserve urethral integrity.

In terms of safety, our findings align with the existing literature indicating that Optilume is generally well tolerated. No instances of urethral perforation, dissection, or procedure-induced erectile dysfunction were observed. This is particularly reassuring given the anatomical proximity of the penile urethra to neurovascular bundles and erectile tissue. The absence of serious complications across multiple studies enhances confidence in the safety profile of this technology and supports its broader adoption in clinical practice.

These findings support future research efforts focused on prospective, randomized trials comparing Optilume to established treatments such as DVIU and urethroplasty, particularly in the penile urethra—a region where the use of Optilume is not yet officially indicated. As a result, patient recruitment for clinical studies in this setting remains especially challenging. However, encouraging results such as those presented here may serve as a starting point and provide the necessary momentum to initiate larger, structured investigations in this anatomically complex and therapeutically underexplored region. Such studies should incorporate objective outcome measures, including uroflowmetry, imaging, or calibration, and should evaluate long-term recurrence rates and cost-effectiveness. At the same time, standardized patient-reported outcome measures (PROMs) would be essential to capture the clinical impact from the patient’s perspective. Our results offer both encouragement and a rationale for continued investigation of this minimally invasive technique in penile strictures.

This study has several limitations. First, the small sample size significantly limits the statistical power and generalizability of the findings. However, given that the use of drug-coated balloon dilation in penile urethral strictures is currently off-label, this limited cohort reflects the real-world clinical caution and regulatory context under which such cases are treated. Additionally, although the methodological design corresponds to a retrospective observational study, the small sample size and off-label nature of the intervention confer characteristics similar to a feasibility study. Therefore, the findings should be interpreted as exploratory in nature.

Second, the absence of a control group prevents direct comparison with standard treatments such as internal urethrotomy or open urethroplasty. Notably, in this series, patients either declined urethroplasty or were not suitable candidates for it and opted for Optilume as a treatment alternative.

Third, the retrospective and non-comparative design introduces potential selection and reporting biases. It is important to note that the study was initiated following unexpectedly positive outcomes observed in a small initial series of patients treated with this approach.

Fourth, no objective functional testing (such as uroflowmetry, cystoscopy, or endoscopy) was systematically performed at follow-up. While this may be viewed as a limitation, it reflects current EAU panel recommendations, which emphasize patient-reported outcomes and discourage routine invasive follow-up in asymptomatic patients. Follow-up in our study, thus, focused on symptom evaluation, patient satisfaction, and absence of retreatment, rather than anatomical assessments.

Finally, correlation analyses between stricture length and treatment outcomes were underpowered and should be interpreted with caution due to the limited sample size.

## 5. Conclusions

The results of this study demonstrate the effectiveness of Optilume as a treatment option for penile urethral strictures. Significant improvements were observed in both lower urinary tract symptoms (IPSS) and quality of life (QoL) scores following treatment, with reductions that reached statistical significance. Notably, these improvements were maintained over a median follow-up period of 16.5 months, during which no re-interventions were required, highlighting the mid-term durability of the treatment.

Although outcomes were comparable between patients with and without prior interventions, those without a history of previous treatment tended to show slightly better results. This suggests that early use of Optilume may offer the greatest benefit, although confirmation will require larger studies.

The stricture length did not appear to influence treatment efficacy within the range evaluated, as no significant relationship was found between stricture length and improvements in IPSS or QoL.

Optilume represents a minimally invasive alternative for managing penile urethral strictures, combining mechanical dilation with the anti-fibrotic effects of paclitaxel. Nevertheless, recent evidence suggests that long-term outcomes in penile strictures may be less durable than in bulbar disease [24]. Therefore, while our results are encouraging, conclusions regarding the role of Optilume in penile strictures should remain cautious until larger, penile-specific prospective trials are available.

From a healthcare systems perspective, Optilume offers logistical advantages; the procedure can be performed in an outpatient or office-based setting, reducing hospital resource use, shortening recovery time, and potentially improving patient satisfaction. However, the initial cost of the device remains a barrier in some contexts, particularly where public reimbursement is not available. Future cost-effectiveness analyses comparing Optilume to repeated DVIU or delayed urethroplasty could provide important insights into its broader adoption.

Patient-reported outcomes should remain central in the evaluation of urethral stricture therapies. While structured instruments such as IPSS and QoL scores provide measurable data, qualitative assessments of urinary comfort, sexual function, and overall satisfaction can capture critical real-world aspects of success. Incorporating standardized patient-reported outcome measures (PROMs) into future trials and registries will be essential to fully characterize the patient experience.

While limited in scale, this study provides a meaningful contribution to the emerging body of evidence supporting the use of drug-coated balloon technology in penile urethral strictures. Given the current lack of official indication for this anatomical location, patient recruitment for clinical trials remains particularly challenging. However, the favorable outcomes reported here may serve as a catalyst for broader clinical adoption and facilitate the design of future prospective, multicenter trials with larger penile-specific cohorts. Such studies will be key not only in confirming the therapeutic value of Optilume in this setting, but also in optimizing patient selection and informing evidence-based treatment pathways. Until more robust data are available, its use should be considered selectively, particularly in patients unfit or unwilling to undergo urethroplasty.

## Figures and Tables

**Table 1:** Demographics and urological medical history of the Patients.

Variable	Mean	Median (Range)
Age (years)	61	59.0 (47–79)
Number of Strictures	1.0	1.0 (1–1)
Number of Prior Treatments	0.6	0.5 (0–2)
Stricture Length (cm)	3.4	3.0 (1.5–8.0)
Follow-up to Jan 2025 (Months)	22.8	16.5 (14–43)
Pre-treatment IPSS	23.1	26 (14–28)
Pre-treatment QoL Score	4.5	5 (4–5)

**Table 2:** Summary of results for IPSS, IPSS QoL, % of improvement, reintervention at baseline and follow-up, with Wilcoxon test; n = 8 patients; *p*-values calculated using Wilcoxon signed-rank test.

Parameter	Baseline	Post-Treatment at Follow-Up (Jan 2025)	Wilcoxon Test
IPSS			
Patients	8	8	
Median (range)	25.5 (14−28)	5.0 (1−17)	*p* < 0.01
% Improvement	–	48.6%	
IPSS QoL			
Patients	8	8	
Median (range)	4.5 (4−5)	1.0 (1−4)	*p* < 0.01
% Improvement	–	50.0%	
Reintervention	–	0/8 (0%)	–
Follow-up, mo	–	Median 16.5 (14−43)	–

## Data Availability

The data presented in this study are available on request from the corresponding author.

## Abbreviations

The following abbreviations are used in this manuscript:

IPSS	International Prostate Symptom Score
QoL	Quality of Life
DVIU	Direct Vision Internal Urethrotomy
EAU	European Association of Urology
DCB	Drug-Coated Balloon
LUTS	Low Urinary Tract Symptoms
Fr	French
PROM	Patient-Reported Outcome Measures
